# Constraining the many-worlds interpretation of computational neuroscience with neurophotonics: a conversation with Gaute Einevoll

**DOI:** 10.1117/1.NPh.10.1.010103

**Published:** 2023-03-07

**Authors:** Anna Devor

## Abstract

Neurophotonics editor-in-chief Anna Devor discusses neurophotonics and computational neuroscience in conversation with Prof. Gaute Einvoll.

In neurophotonics, we often focus – as we should – on the technical aspects of how we interface with the brain. First, we build technologies to measure and manipulate brain structure and function at some spatiotemporal scale. For example, we shape the point spread function of a two-photon excitation beam to optimally overlap with the cell membrane at the focal plane. In parallel, we use molecular engineering to create fluorescent voltage sensors for measuring single-neuron spiking with a millisecond precision and single-neuron resolution. We then apply these neurophotonic technologies to test a specific hypothesis about neuronal “code.”

If we are lucky, an experiment would produce a “Yes” or “No” answer to support or reject the null hypothesis. Most real-world problems, however, do not fall into this category. Rather, the data we acquire require interpretation. This may be due sparse sampling (e.g., a small percent of neurons in a network), an indirect relationship between the measured parameters and the variables of interest (e.g., between the hemodynamic fNIRS signals and the underlying neuronal electrical activity), or other limitations.

This is where computational neuroscience comes to the rescue. Computational modeling translates a problem into a formal, mathematical language where hypotheses can be validated or rejected. Models can also be used to recover “hidden” variables of interest that are not directly measured (e.g., electrical neuronal activity in an fNIRS experiment) and to bridge observations across scale and levels of description. Due to the complexity of brain phenomena, computational neuroscientists usually obtain a family of models or solutions. Constraining this “many-worlds” interpretation of computational neuroscience ideally requires specific kinds of data prescribed by the model. And that, in turn, calls for coordination between the modeling and experimental efforts.

**Figure:**
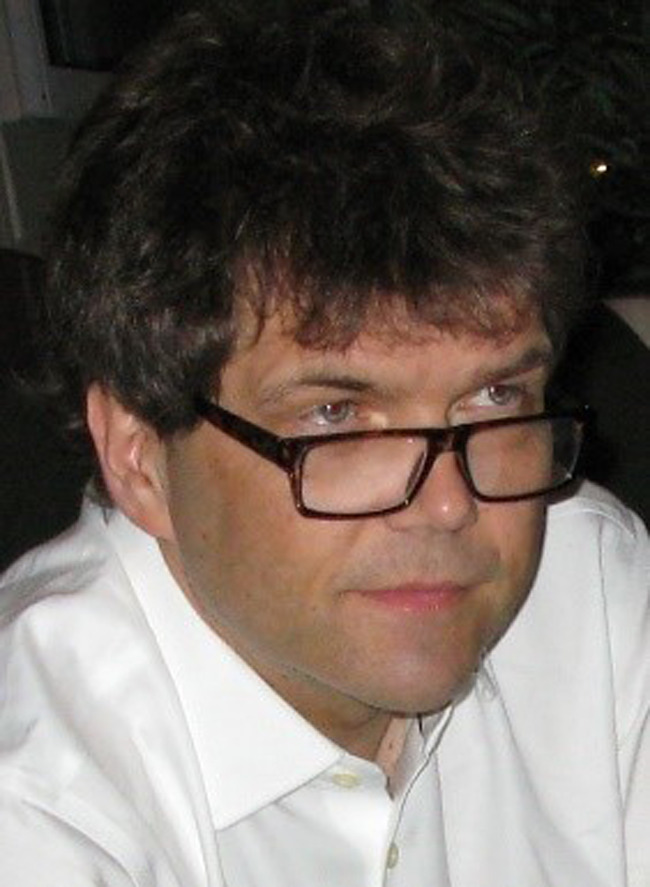
Prof. Gaute Einvoll, Univ. of Oslo and Norwegian Univ. of Life Sciences.

On this note, I’d like to introduce to you Professor Gaute Einevoll who uses methods from physics, mathematics, and computer science to understand how neurons and networks give rise to biological intelligence. A disclaimer: Dr. Einevoll (Gaute) has been my friend and colleague for over 20 years, during which we co-authored over 20 publications. He obtained his PhD in condensed matter physics in 1991 and got introduced to neuroscience at a graduate summer school in Woods Hole. One of the instructors at this summer school was David Kleinfeld, a pioneer in neurophotonics (recent interview, https://doi.org/10.1117/1.NPh.9.1.010401). Yes, it’s a small world.

Today, Dr. Einevoll is a professor of physics at University of Oslo and Norwegian University of Life Sciences. He is a co-leader of the Norwegian node of the International Neuroinformatics Coordinating Facility (INCF) and a partner in the European Union Human Brain Project. Dr. Einevoll is an avid educator and science communicator. He has a podcast titled “Vett og vitenskap med Gaute Einevoll” (“Sense and Science with Gaute Einevoll”) with episodes in Norwegian and English. Dr. Einevoll and co-authors just finished the manuscript for the 2^nd^ edition of the textbook *Principles of Computational Modeling in Neuroscience* that I use as the main text for an undergraduate course I teach at Boston University. Of particular relevance for the topic of this editorial, Dr. Einevoll has contributed two new chapters for this edition, one of them on the modeling of brain measurements. He also has an upcoming new book titled *Electric Brain Signals* – written in collaboration with colleagues in the Oslo research group – that should hit the shelves in 2024.

**Figure:**
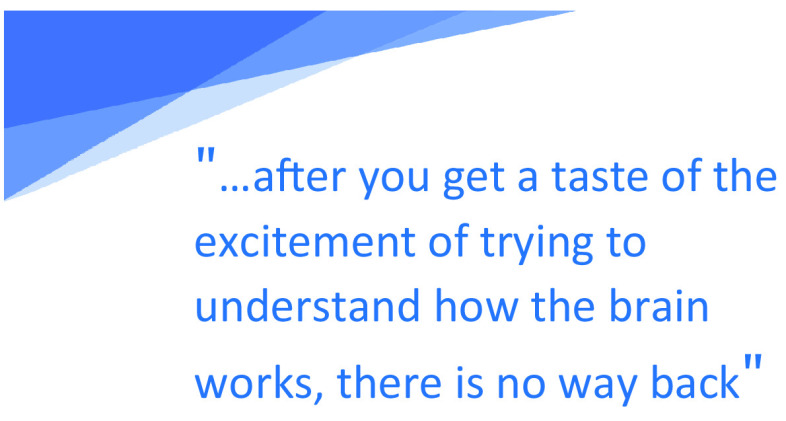


Recently, I asked Dr. Einevoll to share his perspective on the importance of partnership between neurophotonics and computational neuroscience. Below are excerpts from this conversation.

Anna: Let’s start with a personal question, what brings a physicist to neuroscience?

Dr. Einevoll: For me it was accidental. I was a postdoc doing theoretical condensed-matter physics in San Diego in the early 90s where I was introduced to computational neuroscience by another Norwegian, Anders Dale. I realized, like many other physicists have, that my physics toolbox could be put to good use in neuroscience. After all, the brain is a physical system obeying the same well-established laws of physics as dead matter. And after you get a taste of the excitement of trying to understand how the brain works, there is no way back.

Anna: I understand. On the subject of well-established laws of physics, we have a rapidly expanding arsenal of microscopic neurophotonic tools including transparent electronics. This allows seamless integration of optical imaging and optogenetic actuation with electrophysiological recordings in a “Multiphysics” experiment. How can physics-type modeling take advantage of these data?

Dr. Einevoll: Typically, both neurophotonic and electrophysiological data are analyzed by purely statistical means. For example, optical imaging data are often analyzed by correlating measured optical signals with applied sensory stimulation or the behavior of animals. This follows the tradition of Hubel and Wiesel who used electrode recordings to measure receptive fields of neurons in the visual system. Here physics-type modeling can help to understand the biophysical origin of the signal by simulating the whole measurement, for example, simulating intracellular calcium dynamics in neurons to better understand the signal picked up in a two-photon calcium imaging experiment. The topic of our upcoming book *Electric Brain Signals* is on this type of “measurement modeling” for electric and magnetic signals such as spikes, LFP, ECoG, EEG, and MEG.

Another application of physics-type modeling is to compute brain signals for parameter fitting and validation of candidate biophysics-based neuron and network models. This is the traditional physics approach in natural science. With both neurophotonic and electrophysiological data recorded from, say, the same cortical circuit, it is easier to home in on the correct circuit model.

Anna: Let’s talk more about using experimental data for modeling cortical circuits such as a cortical “column” in the mouse sensory cortex. Wasn’t this the main goal of the Blue Brain and now Human Brain Project?

Dr. Einevoll: Yes, our group in Oslo is collaborating with Anton Arkhipov and Christof Koch at the Allen Institute in Seattle to develop what we eventually hope will be a “multipurpose” biophysics-based model for the mouse primary visual cortex. With the term “multipurpose” we imply that the model should be able to account for various types of experimental data under many different perturbations (including optogenetic and sensory stimulation) and brain states. These models will likely be very complex and almost as hard to understand as the real visual cortex. However, it will it be a fantastic starting point for gaining more understanding as it will be a “white box” where all parameters can be changed at will, like a perfect biological testbed.

**Figure:**
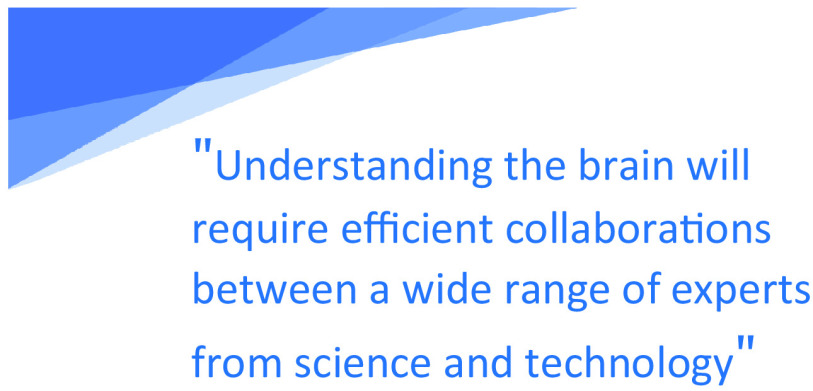


This is similar in spirit to the Blue Brain Project, but a difference, as I understand it, is that we are less “bottom-up” in that a key focus is on fitting of the models to the measurements. This fitting is a challenging undertaking, both for practical and principled reasons. As has been vividly demonstrated in Eve Marder’s group for a much simpler circuit in the crab stomach, there is no unique solution to the parameter fitting problem. So, we have to learn how to fit models to data in such situations with highly degenerate solutions.

The Human Brain Project focuses on developing tools for simulating large-scale networks and allowing for the widespread use of simulation tools like NEURON, NEST and TVB by the broader community. Here our own group contributes with our simulation tool LFPy which can compute electric and magnetic brain signals like LFP, EEG, ECoG, and MEG based on outputs from these network simulators.

Anna: With your focus on brain’s electric signals, you are aware of course of the recent push for high-yield electrophysiological devices with large-scale coverage. In microscopic neurophotonics, we have parallel advances in large-scale imaging technologies, e.g., cell-resolved, volumetric single- and multiphoton calcium imaging. With the rollout of these novel technologies, there is a growing appreciation of sampling biases specific to each measurement modality. Can a physical model help?

Dr. Einevoll: Yes, electrical and optical signatures of cellular activity can be computed by such physical models and can provide direct insights into such biases. As an example, we have already used this approach to study how shapes and amplitudes of spikes depend on the morphology and electrical properties of the neuron (Pettersen and Einevoll, *Biophysical Journal*, 2008).

Anna: We already mentioned optogenetics that is widely used for cell-type-specific manipulation of brain circuits. How do we capture neuronal stimulation or inhibition due to these optical actuators in a biophysical model?

Dr. Einevoll: In neuron models the effect of optical stimulation is included in a direct and natural way since CHR2 is just another ion channel. This makes the simulation of such stimulation easier and cleaner than electrical or magnetic stimulation.

Anna: So, knowing that a project will sooner or later require modeling, at what stage of the project do we (neurophotonics experts) need to coordinate with computational experts?

Dr. Einevoll: I would think that everyone would benefit from having a good understanding of the physical origin of the optical signal that is measured. Often such understanding is aided by physics-type modeling.

If the goal is to use the data to constrain neuron or network models, I think computational experts should be consulted at the outset. In fact, it could be considered whether it is possible to simulate the experiment in a physical model before, or in parallel to, doing the experiments.

Anna: How do you know that a model you have is valid?

Dr. Einevoll: This is not a question that has a simple answer. The validity of a model depends on the required accuracy of novel predictions from the model. There will always be parameter uncertainty in biophysically-detailed models fitted to experiments, that is, there will always be many parameter combinations that make the model fit the data equally well. The goal of the fitting is thus not to identify a unique model, but rather to identify a set of models that are most compatible with available data. And the more data you have to constrain the model, the smaller you can make this set. In fact, multimodal neurophotonic and electrophysiological data can be very useful as these types of data typically offer complementary constraints.

Anna: Can you give an example?

Dr. Einevoll: Sure, let’s consider this scenario (see Fig. 1). If a pyramidal neuron receives an excitatory synaptic input to the apical dendrite, you will measure a sink (negative hump) in the current-source density (CSD), essentially the double spatial derivative of the local field potential (LFP), above the neuron. However, an inhibitory input into the basal dendrite will also give a negative hump in the CSD measured above the neuron. This follows from the properties of the cable-equation description of the neuron. The electrophysiological measurement alone cannot disambiguate these two possibilities.

**Fig. 1:**
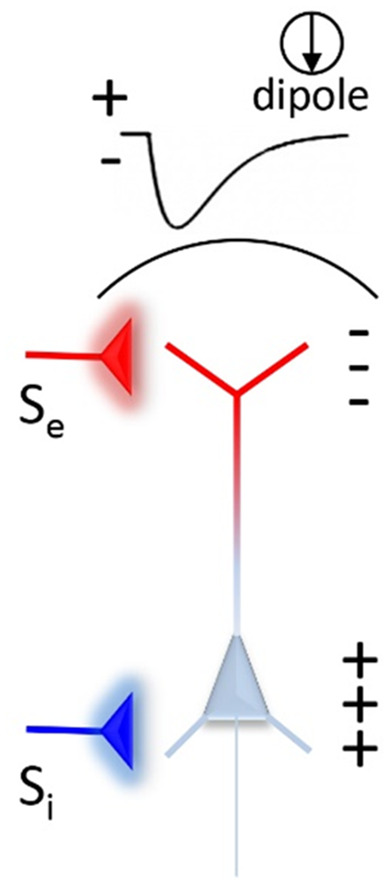
Schematic illustration of the surface potential and current dipole due to excitatory (S_e_) and inhibitory (S_i_) inputs to a pyramidal neuron.

**Figure:**
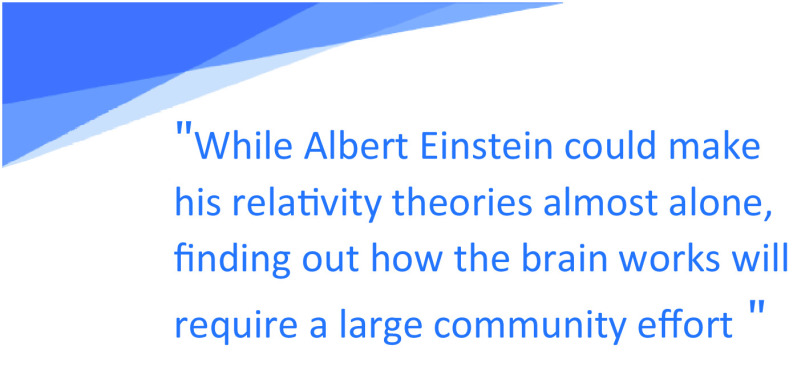


But if we in addition have an optical voltage probe reporting the membrane potential of the apical dendrites, we can distinguish these scenarios. The optical voltage signal reflects local membrane polarization which has opposite signs for excitation and inhibition.

Anna: To conclude on a high note, the BRAIN Initiative has been very successful in generating a wide spectrum of neurophotonic tools motivated by and tailored to the experimental neuroscience needs by encouraging and promoting collaboration between technology and biology experts. What is your view on big team science and the relationship between BRAIN and HBP?

Dr. Einevoll: Being a physicist by training, it is natural to compare with physics. The huge successes in this field have been built on specialization and division of labor between experimental and theoretical researchers, combined with tight collaborations between the groups. Understanding the brain will require efficient collaborations between a wide range of experts from science and technology. Such fruitful collaborations between experts with disparate trainings will require that we strive for a culture of openness and respect. Here there is clearly room for improvement.

Since neuroscience is an information-rich subject spanning many spatial and temporal scales we need big-team projects like BRAIN and HBP to develop the required measurement technology, map out the required neurobiological data, share the data, and build models and computational infrastructure to test candidate ideas against experiments. Together with colleagues in the HBP, I wrote a paper outlining this perspective in case readers are interested.

While Albert Einstein could make his relativity theories almost alone, finding out how the brain works will require a large community effort. As has been said: “It takes a world to understand the brain.”

